# Threat Detection in Nearby Space Mobilizes Human Ventral Premotor Cortex, Intraparietal Sulcus, and Amygdala

**DOI:** 10.3390/brainsci12030391

**Published:** 2022-03-15

**Authors:** Aline W. de Borst, Beatrice de Gelder

**Affiliations:** 1 Department of Biological and Neuropsychology, Faculty of Psychology and Human Movement, Hamburg University, Von-Melle-Park 11, 20146 Hamburg, Germany; 2 UCL Interaction Centre, University College London, 66-72 Gower St., London WC1E 6EA, UK; 3 Brain and Emotion Laboratory, Department of Cognitive Neuroscience, Faculty of Psychology and Neuroscience, Maastricht University, Oxfordlaan 55, 6229 EV Maastricht, The Netherlands; b.degelder@maastrichtuniversity.nl

**Keywords:** peripersonal space, threat, virtual reality, fMRI, visual looming

## Abstract

In the monkey brain, the precentral gyrus and ventral intraparietal area are two interconnected brain regions that form a system for detecting and responding to events in nearby “peripersonal” space (PPS), with threat detection as one of its major functions. Behavioral studies point toward a similar defensive function of PPS in humans. Here, our aim was to find support for this hypothesis by investigating if homolog regions in the human brain respond more strongly to approaching threatening stimuli. During fMRI scanning, naturalistic social stimuli were presented in a 3D virtual environment. Our results showed that the ventral premotor cortex and intraparietal sulcus responded more strongly to threatening stimuli entering PPS. Moreover, we found evidence for the involvement of the amygdala and anterior insula in processing threats. We propose that the defensive function of PPS may be supported by a subcortical circuit that sends information about the relevance of the stimulus to the premotor cortex and intraparietal sulcus, where action preparation is facilitated when necessary.

## 1. Introduction

A long tradition of animal research has shown how the brain monitors the space surrounding the body, referred to as “peripersonal space” (PPS) [1]. PPS was traditionally defined as a fixed physical space surrounding the body. More recently, it was argued that the metrics of PPS change as a function of the behavioral relevance of the stimulus [2]. If we think about PPS in the context of social interactions, body expressions of emotion may be among the most important determinants of behavioral relevance because they signal the need for adaptive action. For example, when walking along the street, we usually ignore other pedestrians as they pass by, but if we notice that one of them is angry, we might need to react. If the angry passerby remains on the other side of the street, it may not prompt any change in our behavior, but if the person were to cross over and approach, the relative importance increases with the diminishing distance and actions to deal with it now need to be considered. At present, there is still relatively little human research integrating questions on the neural basis of PPS with studies of how threatening social expressions are perceived and reacted to [3,4]. Bringing these research domains together raises the question of whether the threat value of an approaching social stimulus impacts the brain system that sustains PPS.

Classical research on PPS has found that bimodal neurons in the periarcuate region of the premotor cortex and the ventral intraparietal area of the monkey brain respond specifically to visual and auditory stimuli in nearby space and to somatosensory stimuli touching the skin [1,5,6,7,8,9]. A large proportion of these multisensory neurons have spatially related visual, auditory and tactile receptive fields that can be anchored to different parts of the body and have congruent response properties [1,7,9]. The multisensory representation of nearby space by these neurons is thought to support the coordination of actions for object manipulation, such as grasping for food. Recent work in monkeys has suggested that the representation of PPS by the premotor cortex and the ventral intraparietal area may have an additional function, namely, to initiate and coordinate defensive behavior toward threat. Cooke et al. [10,11,12] found that microstimulation of neurons in the polysensory zone of the premotor cortex and the ventral intraparietal area evoked complex movements similar to defensive reactions evoked by air puffs. As many of the bimodal neurons in the ventral intraparietal area respond optimally to visual motion on a trajectory toward the tactile receptive field, this led them to propose that these neurons function to detect approaching objects and organize defensive movements [11].

Consistent with the neurophysiological results in monkeys, neuroimaging research has shown that regions in the ventral premotor cortex (vPM) and intraparietal sulcus (IPS) of the human brain encode PPS [13,14,15,16,17]. In PM, both ventral and dorsal regions have been reported to show visuo-tactile multisensory responses to motion stimuli near to or on the body [13,14], while in the IPS, activations were mostly confined to the anterior part of the sulcus [15,16,17,18]. Unimodal “looming” stimuli moving toward the body also activate the vPM and the IPS in conjunction with a more extensive network of brain regions [18,19,20]. The areas in human vPM and IPS do not only show overlapping responses to visual, auditory, and tactile motion stimuli [21], but the posterior parietal cortex also contains aligned visual and tactile topographic maps [15,17]. These results indicate that, similar to the organization in the monkey brain, multisensory areas in the human vPM and IPS integrate information within PPS.

In humans, the coordinated processing of multisensory information has also been suggested to support defensive reactions to objects moving toward the body, of which looming may be an important component [22], and the avoidance of obstacles [15]. Behavioral research has shown that nearby space representations are sensitive to the threat value of looming visual stimuli [23,24]. Threatening stimuli, including spiders and snakes, reduced the perceived time-to-collision of the stimulus compared to non-threatening stimuli, showing that the perceived time of collision is modulated by emotional factors. Moreover, numerous behavioral studies have pointed out that interpersonal emotions influence PPS perception [25,26,27,28,29,30]. These behavioral studies suggest that the behavioral relevance of emotional stimuli indeed changes PPS representations. However, little research has been performed on understanding the underlying neural mechanisms that support these behavioral changes.

So far, it has been challenging to create realistic social threat situations under neuroimaging laboratory conditions, but increased sophistication and accessibility of immersive virtual reality (VR) now makes this feasible [31,32,33]. In this study, we use an immersive VR environment to create a realistic threat situation to build a bridge between two distinct research fields: the neural basis of PPS and emotion perception. Our goal was to find support for the hypothesis that rather than viewing PPS as a fixed physical space surrounding the body, PPS representation in the brain is sensitive to what the stimulus signifies for the appropriate course of action. We investigated if human brain regions underlying PPS representation (vPM and IPS) respond more strongly to approaching stimuli that are behaviorally relevant for actions avoiding body contact, i.e., supporting the proposed “threat detection” function of PPS. Furthermore, we also investigated whether the amygdala (AMG) and anterior insula (aINS) show stronger responses to threatening vs. non-threatening approaching stimuli. The AMG and aINS have been proposed to function as behavioral relevance detectors [34,35,36], and the aINS has also been shown to respond to visual looming [37]. Moreover, both the INS and AMG play an important role in social threat perception and aggression [38,39,40]. We hypothesize that if threat detection is an important function of the brain network that encodes PPS, threatening stimuli will drive vPM and IPS more strongly. This would provide evidence for an equivalent role of PPS in threat detection in humans and monkeys. Additionally, we expect emotion processing regions, especially the AMG, to similarly show increased responses to approaching stimuli signaling threat.

Our results demonstrated that similar to the monkey brain, the interconnected premotor and intraparietal regions that support PPS in the human brain have a role in the monitoring of approaching threatening stimuli in order to initiate avoidance behavior.

## 2. Materials and Methods

### 2.1. Participants

Twelve healthy volunteers participated in this study. Three participants were excluded from the analyses due to excessive head motion, resulting in a total of nine participants. Four of the participants were male (mean age 20 years; range 18–22) and five were female (mean age 21 years; range 19–27). All participants had normal or corrected-to-normal vision and provided their informed consent. Exclusion criteria were the institute’s MRI safety criteria. In addition, we excluded people from participation that were ever a victim of a violent crime (e.g., burglary, assault, etc.). The study was approved by the institutional Ethics Review Committee Psychology and Neuroscience of Maastricht University in accordance with the Declaration of Helsinki.

### 2.2. Stimuli and Materials

**Figure 1:**
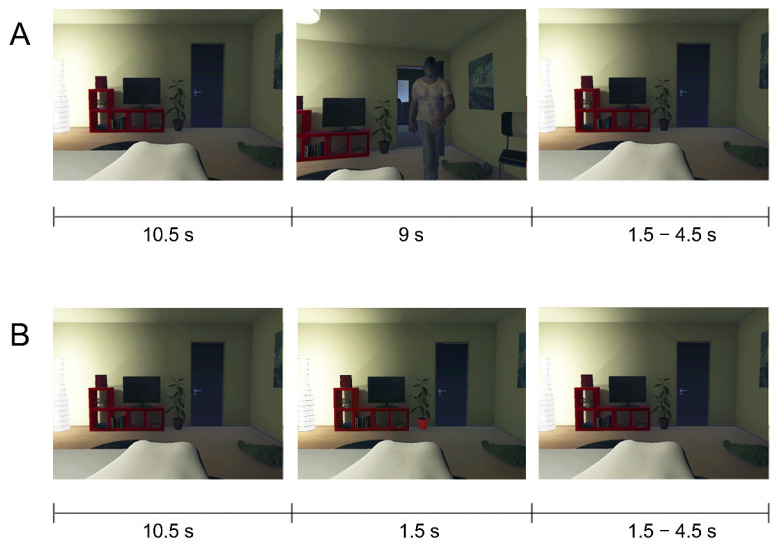
Example of (**A**) an experimental trial and (**B**) an oddball trial.

The stimuli consisted of a VR environment, eight 3D split-screen videos of the VR environment with different events occurring, and four images of the VR environment with an object in a divergent color. The VR scene displayed a bedroom interior from the first-person perspective of a person lying in bed (see Figure 1, left). The 3D videos were split-screen recordings of the VR environment where one of four virtual characters entered the room, and the virtual camera followed the character, similar to a head movement (experimental conditions), or the exact same virtual camera movement occurred as in each of the experimental conditions without the presence of a virtual character (control conditions). The four virtual characters were two threatening (one light-skinned man, one dark-skinned man) and two non-threatening characters (one dog, one child). In the eight different videos (Figure 1A, middle), the following events occurred: (1) a dark-skinned man entered the room and walked toward the bed, stretching his hands forward in a strangling motion (DM), (2) a light-skinned man entered the room and walked toward the bed, stretching his hands forward in a strangling motion (LM), (3) a dog entered the room and walked toward the bed, stretching his head forward (DO), (4) a child entered the room and walked toward the bed, stretching his arms out to be hugged (CH), (5–8) the virtual camera made the exact same movements as in 1–4, but no virtual character appeared (DMM, LMM, DOM, CHM). Each experimental condition had a slightly different camera movement and the control conditions matched each of these movements (e.g., DMM matches DM). We choose these stimuli to simulate non-threatening, naturally occurring events (e.g., child or dog walking in) and threatening events (e.g., burglary). We choose burglary as a threatening event with burglars of different races to eventually compare our results to an ongoing study in South Africa where burglary is a common phenomenon and the most feared threat [41] (Figure 5, p. 17). Each of the videos started with a 10.5 seconds (s) static recording of the room, which served as a baseline image, followed by 9 s of animation, during which the avatar moved from the door to the bed. During the last two seconds of the animation, the video panned to black. The four images displayed the static view of the room in which one object was manually colored in a color divergent from its original color (e.g., black flower pot was colored red) using Adobe Photoshop CS6 (Adobe Systems Software Ireland Ltd, Dublin, Ireland) (Figure 1B, middle).

The VR environment was built in Unity (Unity Technologies, San Francisco, CA, USA). During the training session, the participants viewed the VR scenario using an Oculus Rift DK2 (Oculus VR, Menlo Park, CA, USA), which is a head-mounted display specially designed to view VR. The Oculus Rift has an OLED display with a 960 × 1080 resolution per eye and uses an infrared camera for positional tracking of the headset. Stereoscopic vision was obtained by projecting the stimulus at a slightly different angle to the left and right eye. The video recordings were made from the Unity build using Fraps (Beepa Pty Ltd, Woolloongabba, Australia). The virtual camera movements were performed with recorded mouse movements using Pulover’s Macro Creator (Cloversoft Serviços de Informática Ltda, Sao Paolo, Brazil). During fMRI measurements, the 3D videos were viewed inside the MRI scanner using VisStim MRI-compatible goggles (Resonance Technology, Northridge, CA, USA). The VisStim goggles contain two displays, each with a 600 × 800 resolution, set within a rubber head mount. Similar to the Oculus Rift, stereoscopic vision was obtained by projecting the split-screen video (1600 × 600 resolution) onto the two screens. Additional materials were a threat experience questionnaire, where participants ranked the stimuli according to their threat value (Appendix A, Table A1), and a VR experience questionnaire, where participants rated their experienced presence in the virtual environment and affective and physical reactions on a Likert scale from 1 “Not at all” to 7 “Totally” (Appendix A, Table A2).

### 2.3. Procedure and Task

At the start of the session, participants were informed about the study, filled out the MRI safety checklist, and signed the informed consent form. They were told they could stop with the study at any moment without having to provide an explanation. Next, the subjects were familiarized with the MRI environment. Subsequently, outside of the scanner room, they put on the Oculus Rift and followed auditory instructions by the experimenter to perform several visuomotor exercises (e.g., “turn your head to the left towards the lamp”). During these exercises, the participants looked around in the virtual bedroom environment from a first-person perspective (Figure 1). The camera movements were synchronous with the participants’ head movements in order to increase perceived presence in the virtual environment. After the visuomotor training, the participants closed their eyes (in order to maintain the illusion) and were led to the MRI scanner. During fMRI measurements, the participants were presented with 3D videos of the VR environment (see Stimuli and Materials) where they viewed approaching virtual characters (Figure 1A). The participants were instructed to press a button as fast as possible when they saw an object in the room change color (oddball task; Figure 1B). This oddball task was used to ensure that the participants kept their attention focused on the stimuli. After fMRI measurements, participants filled out the threat experience questionnaire. At the end of the session, participants were debriefed about the study and were asked about how they experienced the scenario and if they were affected by it. Moreover, they were asked to contact the experimenter if they had any reoccurring thoughts or feelings about the experiment afterward. No participant reported being distressed by the experiment or having persisting thoughts or feelings about the experiment.

### 2.4. Design

The experimental design consisted of two factors: avatar presence (avatar, motion-only) and avatar type (DM, LM, DO, CH). The experimental conditions were presented in a slow-event related design with four experimental runs. Each run consisted of 32 experimental trials (4 × 8 videos) and four oddball trials (1 × 4 images). Every trial started with a 3D video, which consisted of 10.5 s of a static room view (baseline), followed by 9 s of animation (experimental condition). The video was followed by a variable baseline period (static room view) of 1500, 3000, or 4500 ms. The oddball trials consisted of 10.5 s of static room view (baseline) 1.5 s of image presentation and were followed by the same type of variable baseline period as described above. The trials with the experimental conditions were presented in a pseudo-randomized order, such that each condition was presented once every eight trials. The length of the baseline period was pseudo-randomized such that each length occurred an equal number of times with each stimulus within the run. The four oddball trials were presented at pseudo-random times during each run, such that each oddball trial was separated from another by at least five trials. The order of the oddball images was randomized.

### 2.5. Data Acquisition

A 3T Siemens MR scanner (MAGNETOM Prisma, Siemens Medical Systems, Erlangen, Germany) was used for imaging. Functional scans were acquired with a multiband gradient echo echo-planar imaging sequence with a repetition time (TR) of 1500 ms and an echo time (TE) of 30 ms. The four functional runs each consisted of 552 volumes comprising 57 slices (matrix = 100 × 100, 2 mm isotropic voxels, interslice time = 26 ms, flip angle = 77°). After the functional runs, high-resolution T1-weighted structural images of the whole brain were acquired with an MPRAGE with a TR of 2250 ms and a TE of 2.21 ms, comprised of 192 slices (matrix = 256 × 256, 1 mm isotropic voxels, flip angle = 9°).

### 2.6. Data Analyses

#### 2.6.1. Functional MRI Pre-Processing

The fMRI data were pre-processed and visualized using fMRI analysis and visualization software BrainVoyager QX version 2.8.4 (Brain Innovation B.V., Maastricht, the Netherlands). Functional data were corrected for head motion (3D motion correction, sinc interpolation), corrected for slice scan time differences, temporally filtered (high pass, GLM-Fourier, 5 sines/cosines), and spatially smoothed using a Gaussian kernel with an FWHM of 4 mm. The anatomical data were corrected for intensity inhomogeneity [42] and transformed into Talairach space [43]. The functional data were then aligned with the anatomical data and transformed into the same space to create 4D volume time-courses.

#### 2.6.2. Behavioral Statistical Analyses

For the threat experience questionnaire (Appendix A, Table A1), we calculated the group mean and standard error of the ranking scores for each stimulus. We performed a Friedman’s ANOVA on the ranking scores, followed by post-hoc Wilcoxon signed-rank tests on the different stimulus pairs (e.g., DM vs. LM, DM vs. CH). The post-hoc tests were corrected for multiple comparisons using Bonferroni at *p* < 0.0083, controlling the FWER at 0.05. For the VR experience questionnaire, we calculated mean responses and the standard errors. The results are reported in Appendix A, Table A2.

#### 2.6.3. Functional MRI Statistical Analyses

We calculated an RFX two-factor repeated-measures ANOVA, with avatar presence and avatar type as main factors, on the whole brain and in pre-defined regions of interest (ROIs). We first calculated a contrast across the brain where we compared avatar vs. motion-only ([DM + LM + DO + CH] > [DMM + LMM + DOM + CHM]) to see which regions responded to the presence of an approaching avatar. The resulting map (t(24) > 3.00) was corrected for multiple comparisons using a false discovery rate (FDR) of 0.05 [44]. To answer our research question we calculated a contrast to compare threatening to non-threatening avatars irrespective of stimulus motion ([DM + LM] > [DO + CM] and [DOM + CHM] > [DMM + LMM]) within regions of interest (ROIs) and across the whole brain. For the ROI-based analyses, anatomical masks for each of the four regions of interest (vPM, IPS, AMG, INS) were manually drawn in each participant’s Talairach-transformed anatomical data on the basis of anatomical landmarks. The ROI results were corrected for multiple comparisons (number of ROIs) using an FDR of 0.05. The whole-brain map (t(24) > 3.09) was corrected for multiple comparisons using cluster-size thresholding at *p* < 0.05, with an initial threshold of *p* < 0.001.

## 3. Results

### 3.1. Behavioral Results

**Figure 2:**
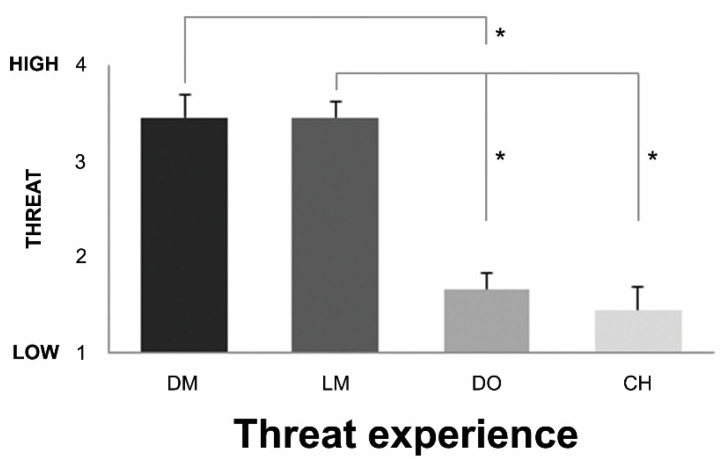
Behavioral responses of VR threat experience questionnaire. The mean (*n* = 9) threat ranking scores of each stimulus and the standard errors are displayed (1 = low threat, 4 = high threat). DM = dark-skinned man, LM = light-skinned man, DO = dog, CH = child. Significant differences between ranking scores (*p* < 0.0083) are indicated with an asterisk *.

The results of the VR threat experience questionnaire (Figure 2) showed a statistically significant difference in threat ranking depending on the stimulus (Friedman’s test, χ^2^(3) = 19.400, *p* = 0.000, Kendall’s W = 0.719). The post-hoc tests (Wilcoxon signed ranks test, with Bonferroni correction applied, resulting in a significance level set at *p* < 0.0083) showed that the dark and light-skinned men were perceived as more threatening than the dog (DM (*M* = 3.44, *SE* = 0.24) > DO (*M* = 1.67, *SE* 0.18), Z = −2.701, *p* = 0.007; LM (*M* = 3.44, *SE* = 0.17) > DO (*M* = 1.67, *SE* = 0.18), Z = −2.724, *p* = 0.006). In addition, the light-skinned man was perceived as more threatening than the child (LM (*M* = 3.44, *SE* = 0.17) > CH (*M* = 1.44, *SE* = 0.24), Z = −2.694, *p* = 0.007). A trend was found for the dark-skinned man to be perceived more threatening than the child (DM (*M* = 3.44, *SE* = 0.24) > CH (*M* = 1.44, *SE* = 0.24), Z = −2.602, *p* = 0.009). We found no differences in threat experience between the dark and light-skinned men (DM (*M* = 3.44, *SE* = 0.24) > LM (*M* =3.44, *SE* = 0.17), Z = 0.000, *p* = 1.000) and the dog and child (DO (*M* = 1.67, *SE* = 0.18) > CH (*M* = 1.44, *SE* = 0.24), Z = −0.577, *p* = 0.564).

The scores on the VR experience questionnaire (see Appendix A, Table A2), rated on a Likert scale from 1 “Not at all” to 7 “Totally”, showed moderate experiences of presence (questions 1, 2, and 4, *M* = 3.78) and moderate affective and physical reactions during the perception of the stimuli compared to reality (questions 3, 5, 6, and 7, *M* = 3.42).

### 3.2. Visual Looming in Nearby Space

**Figure 3:**
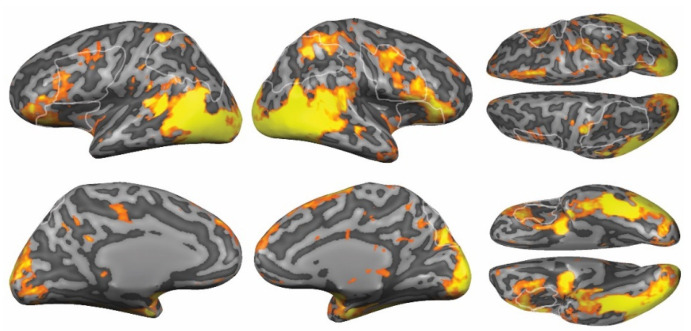
Network in response to the presence of approaching avatars. Results of the RFX ANOVA for avatar presence, FDR < 0.05. The outlines of the maximal probability maps of the ROIs are shown in white.

First, we investigated whether visual stimuli approaching into the participant’s nearby space activated the brain network encoding PPS. The results of the RFX ANOVA analysis (FDR < 0.05) showed the responses to avatar presence in a network across the brain (Figure 3). In accordance with the PPS literature and previous studies [45], we found stronger activation for approaching avatars compared to motion-only stimuli in the PPS network, including ventral PM and IPS, and in temporo-parietal junction (TPJ) and superior parietal lobe (SPL). In addition, we found strong activations in the occipital and occipito-temporal cortex, coding for the visual content, e.g., the face and body of the avatars.

Moreover, we also found stronger activations for avatar presence compared to motion-only stimuli in several regions typically associated with emotion, including INS, orbitofrontal cortex (OFC), AMG, and left cingulate cortex (for a full overview, see Appendix B, Table A3).

### 3.3. Threat Perception in Nearby Space

Our main research question focused on how PPS intrusion by threatening versus non-threatening social stimuli influences brain regions underlying PPS representation and emotion processing. In order to address these questions, we used a contrast to compare threatening to non-threatening avatars irrespective of stimulus motion within our defined ROIs and across the whole brain.

#### 3.3.1. Region-of-Interest Analyses

**Figure 4:**
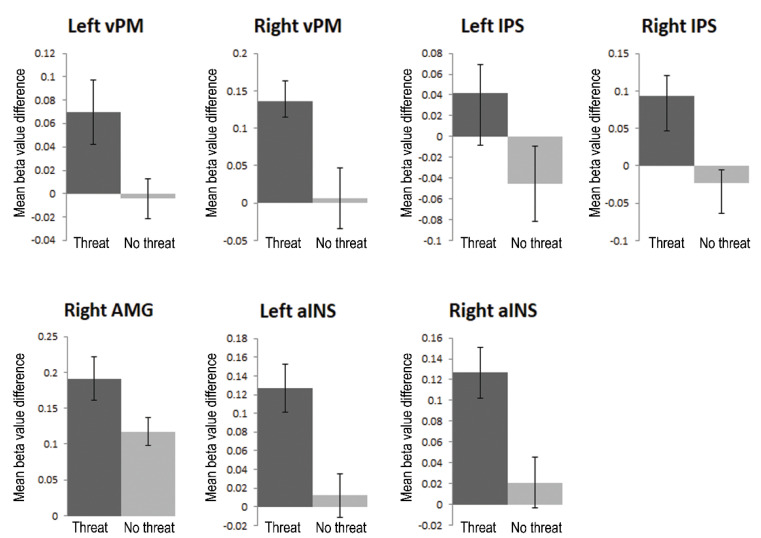
Additional visualizations related to the RFX ANOVA ROI analyses. For each ROI, the plot shows the mean beta value across threat conditions (DM, LM) subtracted with the mean beta value across threat control conditions (DMM, LMM) on the left (dark gray) and the mean beta value across no treat conditions (DO, CH) subtracted with the mean beta value across no-threat control conditions (DOM, CHM) on the right (light gray).

Using RFX ANOVA ROI analyses (FDR < 0.05), we tested whether the main regions of the network that encodes PPS, vPM, and IPS were activated more strongly for threatening than non-threatening avatars, i.e., to show that threat is relevant for the human PPS network. We tested the modulation of PM and IPS by comparing threatening vs. non-threatening stimuli. We found (Figure 4, top) that these regions responded more strongly to threatening than non-threatening intrusion of PPS, in bilateral vPM (LH: t(8) = 2.7, FDR < 0.05; RH: t(8) = 2.5, FDR < 0.05) and bilateral IPS (LH: t(8) = 2.4, FDR < 0.05, RH: t(8) = 2.8, FDR < 0.05). These results indicate that threat is a relevant factor for the encoding of PPS.

Secondly, we specifically addressed our second research question by investigating whether the threatening avatars activated emotion processing regions, e.g., AMG and aINS, more strongly than the non-threatening avatars. We found (Figure 4, bottom) stronger activation for threatening than non-threatening avatars in right AMG (t(8) = 2.6, FDR < 0.05) and bilateral aINS (LH: t(8) = 2.7, FDR < 0.05; t(8) = 2.7, FDR < 0.05), confirming increased responses in emotion processing regions for the threatening avatars. We found no significant difference in left AMG (t(8) = 1.4, FDR > 0.05).

#### 3.3.2. Whole-Brain Analyses

**Figure 5:**
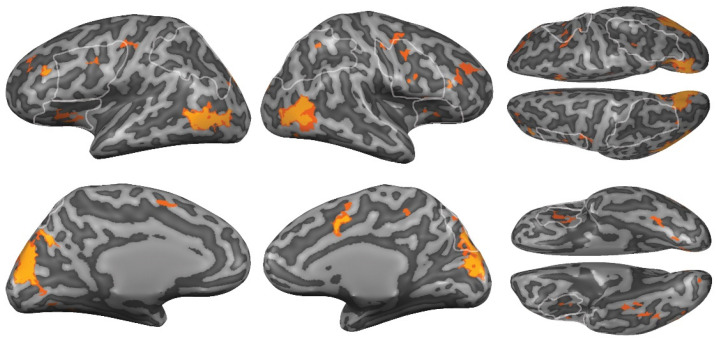
Network in response to presence of threatening vs. non-threatening avatars. Results of the RFX ANOVA threat perception contrast in yellow (initial threshold = 0.001, *p* (corrected) < 0.05), overlaid for display purposes on results with initial threshold = 0.005, *p* (corrected) < 0.05, in orange. The outlines of the maximal probability maps of the ROIs are shown in white.

In order to investigate whether any additional brain regions differentiated between threat and no-threat conditions, we ran a whole-brain RFX ANOVA using the same contrast (*p*(corrected) < 0.05). We found a network (Figure 5 and Appendix B, Table A4) that showed stronger responses to threatening compared to non-threatening avatars in the left middle frontal gyrus (MFG), right anterior cingulate cortex (ACC), bilateral extrastriate body area/MT+, and bilateral cuneus. We found no regions that showed stronger activation for the non-threatening avatars.

## 4. Discussion

The aim of this study was to investigate whether the encoding of PPS in the human brain by vPM and IPS performs a function in detecting approaching stimuli that are behaviorally relevant for actions avoiding body contact, i.e., supporting the proposed defensive function of PPS. We approached this question from a novel perspective within an ecologically valid context using VR. Our results showed that vPM and IPS indeed responded more strongly to approaching fear-evoking stimuli, and that threat was also signaled by the AMG and aINS.

### 4.1. Defensive Behavior in Peripersonal Space

Similar to neurophysiological work in monkeys [46], behavioral experiments have provided evidence for a defensive function of PPS in humans. For example, the boundary of PPS is reduced when faced with threatening objects and sounds [23,24,47,48] or threatening individuals [25,26,28,29]. Visual threats in PPS also heighten physiological responses [49,50] and elicit faster reaction times to tactile stimuli [51,52]. These behavioral results showed that PPS representation is influenced by threatening properties of the stimulus and that it facilitates fast responses to threat. However, so far, there has been little neuroimaging evidence for the involvement of human vPM and IPS in threat detection in PPS. In this study, we revealed how the brain may facilitate these fast behavioral responses.

Our results showed stronger neural responses to threatening looming stimuli in vPM and IPS. Previous electrophysiological research by Vagnoni et al. [53] found that several different electroencephalography (EEG) sites were modulated by the threat value of the approaching animals, including increased alpha and high gamma desynchronization over respectively occipital-parietal and occipital-central sites. Their results suggested an interaction between the threat value of a stimulus and action preparation in the sensorimotor cortex. However, given the limited spatial resolution of EEG, a more detailed account of the involved brain network was not possible. Here, we showed how brain activity increased in response to a nearby visual threat, not only in the human posterior parietal cortex but also in vPM. This indicates that these regions do not only respond to looming and tactile stimuli [17,37,54] but are also modulated by the threat value of the stimulus. Moreover, previous research suggested that subcortical structures might form an important link between emotional value processing and the visual perception of looming [37,53]. Our results provided evidence for this hypothesis by showing that subcortical structures, such as the AMG, and other regions known for their role in emotion and relevance processing, such as the aINS, similarly respond to the threat value of the stimuli. Together these results suggest a mechanism for how behaviorally relevant stimuli in proximity of the body, such as approaching threat, might elicit action preparation.

The premotor cortex and the posterior parietal cortex play, among others, an important role in action planning and preparation. Clearly, not all planned actions have the same importance for the organism. In case of threat, actions to avoid bodily contact should be prioritized and executed quickly. Monitoring of the space surrounding the body supported by vPM and IPS should facilitate the fast detection and response to possible threats. Lloyd et al. [35] showed that painful versus non-painful stimulation of a rubber hand in a congruent location with the real hand increased brain activity in regions including the posterior parietal cortex and aINS. This suggests that the threat value of the visual stimulus affects the planning of protective body movements. However, they did not find any evidence for threat modulation in premotor regions. Here we showed that responses in both IPS and vPM are enhanced when a visually looming stimulus is threatening. This shows that, together with other visual areas (see Figure 5), the body-part centered encoding of visuospatial information in nearby space by human vPM and IPS is sensitive to the threat value of the information.

We also found that regions sensitive to affective properties and behavioral relevance discriminated between threatening and non-threatening looming stimuli. The AMG has been proposed to fulfill an integrative signaling function for stimuli that incite action [19,34,36]. Especially forward-moving looming stimuli induce an increased sense of threat [22], for which the AMG is particularly sensitive [19,55,56]. The AMG is also well known for its role in emotion processing [19,55,56,57,58] and rapid perception and response to fear [59]. It receives input from other subcortical structures, such as the superior colliculus and the pulvinar, with which it forms a close network to process visual emotions [19,55,56,57,58,60,61], including threat [62]. Recently, it has been shown that the basolateral sub-division of the AMG supports active escape from nearby threat through connectivity to the central AMG [63], and the AMG has been shown to be responsive to the threat value of a stimulus depending on the threat’s movement path [56]. Our findings are in line with these results showing that the AMG signals relative importance depending on the threat value of the approaching stimulus. Moreover, we observed that threatening compared to non-threatening stimuli activated the right but not the left amygdala. It is not yet well understood what processes may underlie observed patterns of laterality and hemispheric specialization during emotional processing [64,65]. Recent studies propose moving away from a model of general hemispheric specialization in emotion processing and instead suggest that different aspects of the emotion generation process may have their own distinct lateralization patterns [66]. These hypotheses require further investigation, including studies detailing the role of the different subnuclei of the AMG, as these have specific functions in tuning behavioral reactions to emotional signals. In further relation to emotion and salience processing, we also found that the aINS responded more strongly to threatening stimuli in PPS. The aINS has been suggested to support judgments on the time to the collision of approaching objects in PPS [37]. It has also been shown to play a role in (motivational) affect and has functional pathways to the AMG and the somatosensory cortex [67]. Given that these affective and motivational regions were preferentially responsive to the threatening stimuli, we suggest that they may provide information about the relevance of the stimulus to vPM and IPS to facilitate or inhibit responses.

### 4.2. Behavioral Relevance

In considering the defensive function of PPS, one may ask whether the demonstrated effects are specific to threatening stimuli or whether they apply more generally to a large variety of behaviorally relevant stimuli. Both in the monkey and the human PPS literature threat detection has been singled out as a specific function of PPS monitoring. However, enhanced responses in the human PPS network have not only been found for threatening stimuli. Holt et al. [18] showed that neutral social stimuli also impact PPS representations in the human brain. They found that the vPM and dorsal IPS responded more strongly to looming face stimuli moving toward vs. away from the participant, while this was not the case for cars or spheres. These results indicate that social stimuli are more behaviorally relevant than objects and therefore also enhance responses in vPM and IPS. Behavioral relevance in relation to the PPS representation might possibly be seen as a gradient, with increasing levels of relevance evoking increasing levels of activation in the brain network (see also [2]). For monitoring the space surrounding our body and preparing appropriate responses, neutral social stimuli may be more relevant than neutral objects (as shown by [18]), while threatening social stimuli are more relevant than neutral social stimuli (as shown in this study). However, since our study focused specifically on social threat (which has both high relevance and high salience), unequivocal neuroimaging evidence for the behavioral relevance function of PPS is still lacking. Future neuroimaging research could address this question by comparing neural responses to behaviorally relevant and non-relevant stimuli in the PPS network while controlling for saliency.

### 4.3. Naturalistic Approaches

When studying the neural basis of (social) threat, the experienced behavioral relevance is also largely depending on the type of stimulus that is presented to the participants. Dynamic, moving, and realistic stimuli, such as possible in VR, videos, or mirrors to view the own body, all have been successfully implemented to create a sense that a threat is near the participant’s body [16,25,49,56,68,69]. Virtual reality is particularly suitable for social scenarios, as this creates an environment where participants can see and experience other people while remaining inside the MRI scanner bore. Virtual embodiment training gives participants the sensation that the displayed virtual body belongs to their body through visuo-motor synchrony. By combining this training with the presentation of moving 3D stimuli in the MRI scanner, participants can experience a sensation of “being there” in the virtual environment [70]. The benefit of this sensation is that the brain responses during the VR experience are more comparable to how the brain reacts to a similar situation in reality. This could also be tested more explicitly in follow-up studies by comparing groups with and without embodiment training to understand how this impacts the brain responses to social threat (similar to the work of [45]). As the VR experience is very different from watching static images appear on the screen, it also comes with additional considerations. First, although participants are aware that the stimulus is not real and suspend disbelief (similar to watching a movie), the impact of the stimulus on the participants should be carefully considered and monitored. Secondly, the immersive nature of VR might evoke unexpected reactions from the participant. When a virtual character suddenly comes very close, participants may intuitively move their heads away, causing motion artifacts. Finally, for social stimuli, in particular, conversations with participants in other VR studies [45,71] have taught us that participants may experience an unknown approaching virtual character as mildly threatening, even those that display a neutral emotion. The 3D environment gives participants the sensation that these characters come very close to them and invade their personal space. Therefore, in this study, we chose characters that are inherently less threatening, e.g., a child, to serve as a neutral control. Clearly, these stimuli also differ in other aspects from the threatening characters. Therefore, the results of this study should be further verified and compared in follow-up studies using different types of virtual characters as control conditions, e.g., adults displaying a neutral emotion. Moreover, given the exploratory nature and technical challenges of this study, it had a relatively low number of participants. Therefore, replication or follow-up studies could further validate the results of this study using a higher number of participants.

## 5. Conclusions

Our results demonstrated that, similar to the monkey brain, the interconnected premotor and intraparietal regions that support PPS in the human brain have a role in the monitoring of approaching threatening stimuli in order to initiate avoidance behavior. We propose that this defensive function of PPS is supported by a subcortical circuit that sends information about the stimulus’ relative importance to aINS and further to PM and IPS, where action preparation in body-centered coordinates is facilitated if necessary.

## Data Availability

The data presented in this study are available on request from the corresponding author. The data are not publicly available due to privacy restrictions.

## Appendix A

**Table A1:** Threat experience questionnaire. The questionnaire was administered electronically. Still images of the four conditions were shown to the participants, and they ranked them according to perceived threat level. The order of the images and names in the table were randomized. The table was set such that it was only possible to provide one response per row and column. An example response is shown (x).

Below You See Stills from the Events You Observed in the Scanner. Please RANK These Events on Perceived Threat, from Low (1) to High (4) Threat.				
	1 (Low Threat)	2	3	4 (High Threat)
Man A				x
Child	x			
Dog		x		
Man B			x	

**Table A2:** Questionnaire related to presence and experiences during the perception of the virtual scenarios. Questions 1, 2, and 4 measured the sense of presence and questions 3, 5, 6, and 7 measured affective and physical reactions in the virtual environment compared to reality. Left column: Questions. Right column: Mean responses and their standard errors. Reponses were given on a Likert Scale from 1 to 7 (1 = “Not at all”, 7 = “Totally”).

Questions	Mean Response and Standard Error
1. To what extend did you feel as if you were in the room and lived the situation as if it were real?	4.00 ± 0.74
2. Although you knew you were not there, to what extent did you have the illusion as if you were in the room?	3.89 ± 0.70
3. To what extent did you think things like “I know this isn’t real”, but then surprisingly finding yourself reacting as if it was real?	3.67 ± 0.74
4. To what extent was your sense of being in the room stronger than your sense of being in the scanner?	3.44 ± 0.58
5. To what extent were your emotional responses during the events in the room similar to a real situation?	3.22 ± 0.72
6. To what extent were the thoughts that you had during the events in the room similar to a real situation?	3.33 ± 0.79
7. To what extent were the physical responses that you had during the events in the room similar to a real situation?	3.44 ± 0.84

## Appendix B

**Table A3:** List of significantly activated regions of the RFX ANOVA for avatar presence (t(24) > 3.00, FDR < 0.05), including peak voxel coordinates and their cluster size.

Avatar Presence				
Region	Peak Voxel Coordinates			Cluster Size
	x	y	z	
Left inferior frontal gyrus	−47	21	40	6545
Left inferior frontal gyrus	−45	41	4	3442
Right inferior frontal gyrus	55	27	24	6033
Medial superior frontal gyrus	9	9	64	9645
Anterior medial superior frontal gyrus	−3	43	40	14,724
Left premotor cortex	−41	9	52	3141
Right premotor cortex	37	−5	40	6244
Left insula	−41	21	−4	10,745
Right insula	51	23	6	11,803
Left superior parietal lobe	−55	−57	30	2255
Left intraparietal sulcus	−35	−43	52	3072
Right intraparietal sulcus	29	−53	48	8015
Right precuneus	17	−79	34	4539
Right supramarginal gyrus	47	−37	16	6454
Left lateral sulcus	−55	−41	16	1281
Left mid cingulate sulcus	−15	−21	38	1186
Medial posterior cingulate sulcus	−1	−47	24	2275
Left parahippocampal gyrus	−35	−7	−20	2160
Right parahippocampal gyrus	37	−3	−24	2002
Left occipito-temporal gyrus	−37	−45	−10	7223
Right occipito-temporal gyrus	37	−41	−14	7618
Left middle occipital gyrus	−45	−69	−4	18,119
Right middle occipital gyrus	41	−67	0	18,492
Left middle occipital gyrus	−15	−99	6	18,119
Right middle occipital gyrus	13	−97	4	18,492
Left inferior occipital gyrus	−43	−81	−2	11,367
Right inferior occipital gyrus	41	−75	−6	13,711
Left posterior cingulate sulcus	−53	−41	20	1979
Left amygdala	−25	−3	−12	2745
Right amygdala	17	−5	−10	2590
Left pulvinar	−19	−25	−2	1638
Right pulvinar	15	−25	2	1876

**Table A4:** List of significantly activated regions of the RFX ANOVA for threat perception (t(24) > 3.09, FDR < 0.05), including peak voxel coordinates and their cluster size.

Threat Perception				
Region	Peak Voxel Coordinates			Cluster Size
	x	y	z	
Left middle frontal gyrus	−39	29	32	180
Right anterior cingulate cortex	9	3	40	167
Left middle occipital gyrus	−49	−77	6	2302
Right middle occipital gyrus	39	−63	8	2941
Left cuneus	−7	−81	24	5965
Right cuneus	1	−79	14	6149
